# A suppurative thyroiditis and perineal subcutaneous abscess related with aspergillus fumigatus: a case report and literature review

**DOI:** 10.1186/s12879-018-3617-4

**Published:** 2018-12-27

**Authors:** Jiaying Tan, Jun Shen, Yong Fang, Liping Zhu, Yang Liu, Ye Gong, Hechen Zhu, Zupeng Hu, Gang Wu

**Affiliations:** 1 Department of Critical Care Medicine, Huashan Hospital, Fudan University, No. 12 Middle Urumqi Road, Shanghai, 200040 People’s Republic of China; 2 Department of Infectious Disease, Huashan Hospital, Fudan University, No. 12 Middle Urumqi Road, Shanghai, 200040 People’s Republic of China; 3 Institute of Antibiotics, Huashan Hospital, Fudan University, No. 12 Middle Urumqi Road, Shanghai, 200040 People’s Republic of China

**Keywords:** *Aspergillus fumigatus*, Systemic lupus erythematosus, *Aspergillus* thyroiditis, Caspofungin, Voriconazole, Diagnosis

## Abstract

**Background:**

Invasive aspergillosis is a complication in immunocompromised patients and commonly detected in patients with hematological malignancies, which mostly affect the lungs. Because of its high iodine content, rich blood supply and capsule, the thyroid is considered to be less prone to microbial invasion thus most infectious thyroiditis cases are caused by bacteria. However, a few case reports have described thyroid gland aspergilloses, most of which were due to disseminated invasive aspergillosis.

**Case presentation:**

We first report a case of thyroid gland and subcutaneous labium majus aspergillosis in a Chinese patient who received long-term glucocorticoid treatment for systemic lupus erythematosus (SLE) and lupus nephritis, and then we reviewed 36 articles describing similar aspergillus infections in 41 patients.

**Conclusion:**

We included 29 cases of diagnosed aspergillus thyroiditis and analyzed clinical findings, treatments and outcomes to provide clinical information for diagnosis and prognosis of thyroiditis caused by *Aspergillus fumigatus*.

## Background

Aspergillus fumigatus is the most common form of aspergillus infection in humans, accounting for 70–80% of these infections [1]. Invasive aspergillosis is an increasingly frequent opportunistic infection in immunocompromised patients such as those with an organ transplant, hematological malignancy, those receiving certain types of chemotherapy, patients infected with human immunodeficiency virus, and other types of immunosuppression therapy [2, 3]. Most often through aerosolizing, aspergillus spores first colonize the respiratory tract and related structures such as the nasopharyngeal and facial sinuses. Further immunosuppression markedly increases the risk for invasive disease characterized by tissue invasion and secondary bloodstream dissemination [4]. The majority of thyroid aspergillosis cases are caused by disseminated invasive aspergillosis and are frequently diagnosed postmortem since they can be apparently symptomless or the clinical appearance is complicated by their comorbidities [5].

## Case presentation

**Fig. 1:**
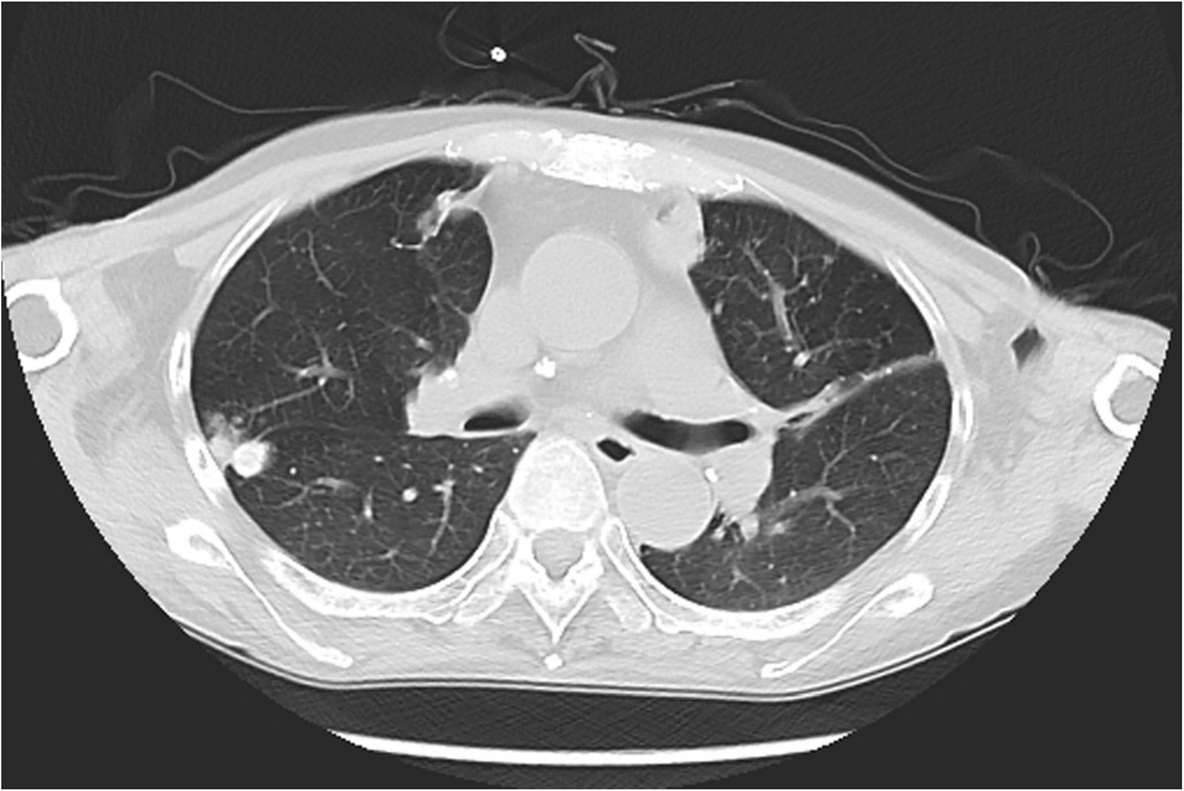
Chest computed tomography showed the bilateral patches in the lungs of the patient as opacities in the middle and lower lobes, multiple shadows of fibrotic streaks, high-density nodules, and mediastinal calcification of lymph nodes

**Fig. 2:**
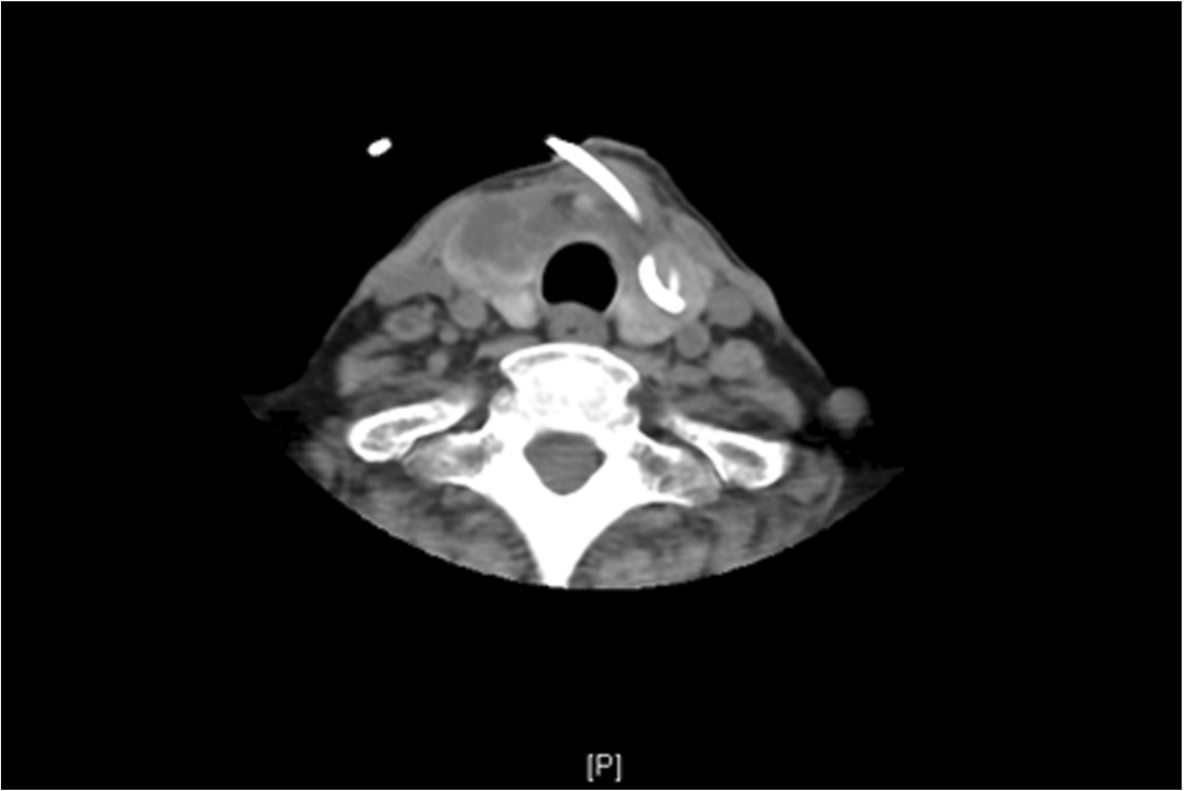
Neck computed tomography revealed effective drainage of the left lobe of the thyroid gland while abscesses remained in the right lobe

A 56-year-old female patient was transferred to our department of critical care medicine, Huashan hospital in Shanghai in June 2016 after she received treatment in a local hospital for productive cough, tachypnea and respiratory distress. She complained of recurrent fever and asymmetric edema of the lower extremities for over 1 month, as well as painful swelling both in the thyroid and labium majus for 2 weeks. In the previous hospital, due to the finding of multiple bilateral cysts which were palpable nodules in her thyroid gland by ultrasound examination, a left lobe thyroid puncture and drainage had been conducted and an aspergillus fumigatus infection was detected. She had a history of systemic lupus erythematosus (SLE) and lupus nephritis for 8 years, and received prednisone treatment for these diseases. But from November 2015, prednisone was switched to methylprednisolone, and hydroxychloroquine has been added because of lupus nephritis aggravation, and tacrolimus has also been added to the medications in the following month. She was also diagnosed with renal hypertension and diabetes induced by steroids, and received antihypertension and antihyperglycemic therapy. She had no history of pulmonary diseases such as chronic obstructive pulmonary disease (COPD), asthma, or any repeated infections, and had no addiction to drugs, smoking or alcoholism. Previous examinations showed no evidence of neutropenia. The ratio of CD4/CD8 was 0.33. Only one aspergillus test was positive in repeated sputum cultures. The galactomannan aspergillus antigen and culture tests in BALF were negative, so were blood and urine cultures including fungi. Our chest computed tomography (CT) imaging revealed bilateral patchy lung opacities in the middle and lower lobes, along with multiple shadows of fibrotic streaks, high-density nodules and mediastinal calcification of lymph nodes (Fig. 1). The diagnosis of pulmonary infection was established, and pathogen was highly suspected of aspergillus according to the previous finding of thyroid puncture and drainage. An ultrasound examination showed thrombosis in the bilateral femoral veins and popliteal veins. In addition, a 51 × 16 mm hypoechoic lesion was detected in the left subcutaneous perineal region**.** We continued voriconazole therapy in a standard treatment dose (200 mg twice a day), but her body temperature was still up to 37.6 °C intermittently. Her white blood cells were 15.61 × 10^9^/L (neutrophils 90.8% and lymphocytes 5.4%), hemoglobin was 93 g/L, and platelets were 295 × 10^9^/L. Except hyperglycemia, proteinuria, and hypoproteinemia, other routine laboratory tests were unremarkable, which including thyroid hormone levels. A neck CT showed findings consistent with a fluid collection in the right thyroid lobe **(**Fig. 2). Cultures of aspirated purulent fluid showed aspergillus fumigatus growth, which was obtained from fine needle aspirations in both thyroid and perineum. Five days after being transferred to our hospital, the patient’s thyroid drainage tube was removed because no further fluid was drained out. We continued the voriconazole dose 400 mg per day as anti-aspergillosis therapy with 16 mg methylprednisolone and 400 mg hydroxychloroquine per day as immunosuppressive therapy, along with a therapeutic 4100 iu q 12 h dose of nadroparin calcium. The patient’s fever was relatively controlled and white blood cells decreased to 10.74 × 10^9^/L (neutrophils 91.7%, and lymphocytes 4.7%). Lesions in the thyroid and subcutaneous labium majus became significantly smaller, and the pain was greatly relieved. On the eighth day of hospitalization, the symptoms had improved and the patient was discharged from our hospital. She continuously took voriconazole orally (400 mg per day) for 6 months, combined with caspofungin for the initial 2 weeks (first day 70 mg, then 50 mg per day). After 1 month of antifungal treatment, she was afebrile and all the clinical symptoms were relieved. The patient is on a follow-up for 1 year and has been free of aspergillosis for 6 months. Hydroxychloroquine treatment ceased in April 2017, and methylprednisolone dose was reduced in a tapered manner.

## Review of the literature

**Fig. 3:**
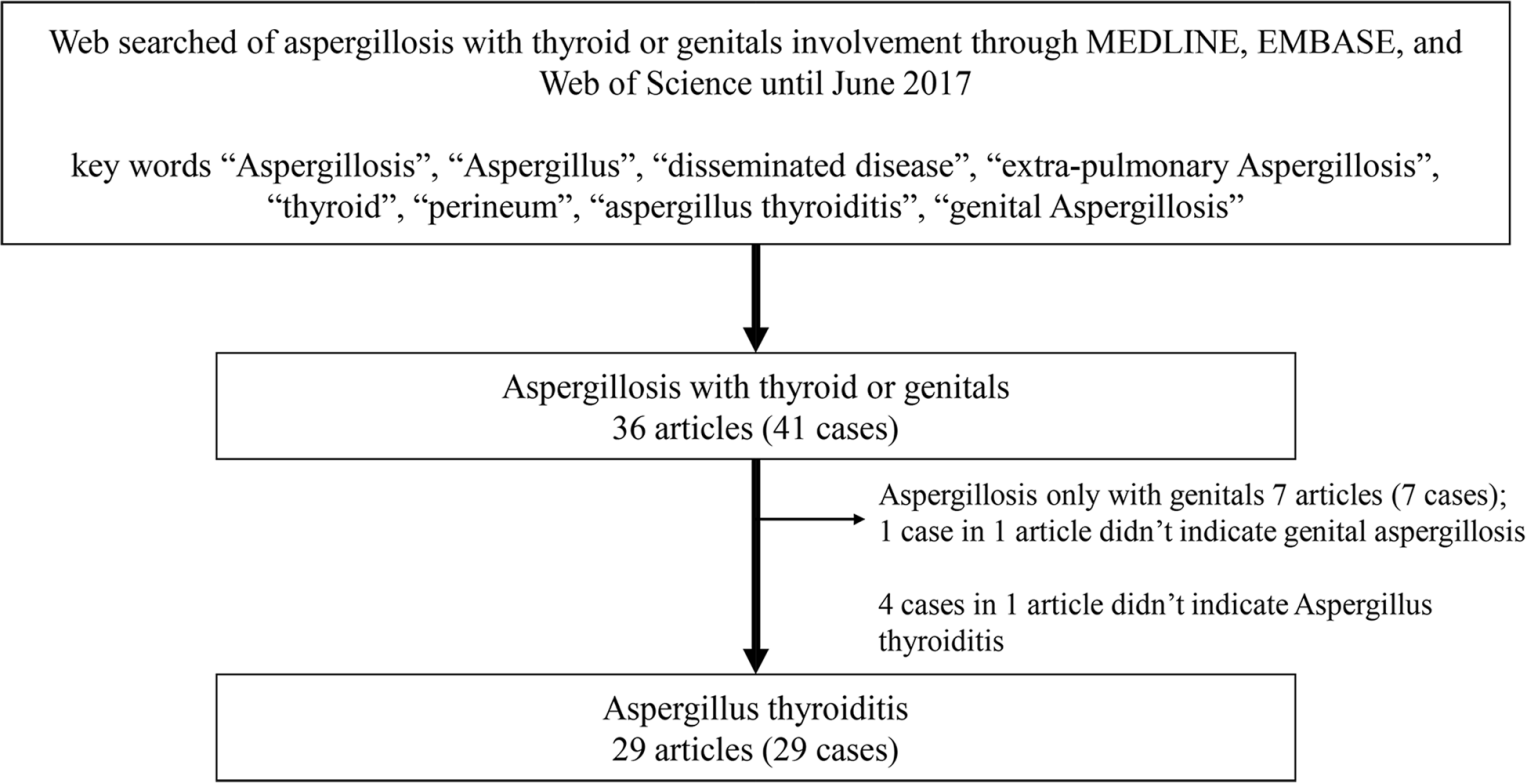
The web search results of publications about aspergillus thyroiditis

To explore thyroid or genital involved invasive aspergillosis in clinical practice, we collected the English literature up to June 2017 for all reports of aspergillosis with thyroid or genital involvement through searches of MEDLINE, EMBASE, and Web of Science. Case reports of neonate or infant patients were excluded because of the immaturity of neonatal skin barrier and immune system. Key words were “aspergillosis”, “aspergillus”, “disseminated disease”, “extra-pulmonary aspergillosis”, “thyroid”, “perineum”, “aspergillus thyroiditis”, and “genital aspergillosis”. Finally, we found 36 articles describing aspergillus infections in 41 patients, from which 7 articles (7 patients) were diagnosed only with genital aspergillosis and 4 cases in an article [6] did not indicate aspergillus thyroiditis. A total of 29 articles describing 29 cases of diagnosed aspergillus infections involving thyroid gland were included for further analysis (Fig. 3).

Most of the aspergillus thyroiditis cases were diagnosed at the age of 11–30 year old (12 cases) [7–18] and 7 cases were over 60 years old [3, 19–24]. 13 of 29 patients had primary infection in lung or airway (44.8%) and primary aspergillus thyroiditis was reported in 12 cases. Except for 1 case [25], all the other patients had critical underlying diseases: 9 cases (29.0%) of organ transplantations [7, 10, 15, 19, 21, 22, 26–29]; 11 cases (35.5%) of hematologic malignancy diseases including 2 cases of acute myelocytic leukemia (AML) (6.5%) [6, 7], 2 cases of acute lymphocytic leukemia (ALL) (6.5%) [17, 30], 3 cases of non-Hodgkin’s lymphoma (NHL) (9.7%) [11, 19, 31] and 4 cases (12.9%) of myelodysplastic syndrome (MDS) [14, 20, 31, 32]; 1 case suffered the acquired immune deficiency syndrome (AIDS) [33]; Another comorbidity was SLE in 5 patients (16.1%) [8, 12, 13, 16, 18] (Table 1).

**Table 1:** Epidemiology, clinical findings, and diagnostic methodology of aspergillus thyroiditis

Epidemiology												
*n* (%)	Age (year-old)							Primary aspergillus locations				
	11-30		31-60		61-78			Thyroid gland			Lung/ airway	
	12 (41.4)		10 (34.5)		7 (24.1)			12 (41.4)			13 (44.8)	
Comorbidities	Transplantation	SLE	MDS	NHL	ALL	AML	AIDS	Chronic granulomatous disease	Polymyalgia	Skin disease	Diabetes	No comorbidity
*n* (%)	9 (29.0)	5 (16.1)	4 (12.9)	3 (9.7)	2 (6.5)	2 (6.5)	1 (3.2)	1 (3.2)	1 (3.2)	1 (3.2)	1 (3.2)	1 (3.2)
Clinical findings												
Symptoms	Fever	Dyspnea^a^	Dysphagia		Neck pain		Neck erythema/ warmth		Tachycardia	Cough	Phlegm	Neurological sign
*n* (%)	25 (86.2)	15 (51.7)	2 (6.9)		14 (48.3)		4 (13.8)		8 (27.6)	5 (17.2)	3 (10.3)	9 (31.0)
Manifestation of thyroid	Enlargement	Nodule	Hemorrhage		Thyrotoxicosis		Euthyroid sick syndrome			Hyperthyroidism	Hypothyroidism	Euthyroidism
*n* (%)	22 (75.9)	10 (34.5)	2 (6.9)		12 (41.4)		1 (3.4)			13 (44.8)	2 (6.9)	6 (20.7)
Diagnostic methodology												
	Imaging				Culture					Others		
	Scintigraphy	Thyroid-CT/ Ultrasound	Lung-X ray/ CT		Thyroid punctured liquid/ swab/ tissue		Sputum/ airway secretion	Blood	Urine/ body fluid/ other tissue	Galactomannan test	Histological test	Fine needle aspiration
Positive, *n* (%)	6 (85.7)	18 (100)	17 (68)		12 (85.7)		6 (33.3)	1 (9.1)	5 (41.7)	5 (55.6)	14 (93.3)	13 (86.7)

^a^including dyspnea/ shortness of breath/ respiratory insufficiency/ airway compromise

### Diagnostic methods and symptoms of patients

Nine patients presented with combination infections, which included bacteria, virus, non-aspergillus fungus. Fever, dyspnea, and thyroid enlargement were the most common presenting symptoms. Dyspnea was the most serious presentation in 15 patients [6–8, 11, 14, 16–18, 20, 21, 24, 25, 29, 31, 32] and caused airway obstructions in 3 patients, which led to 2 deaths [11, 20]. Dysphagia was noted in 2 patient [31, 32]. Based on the laboratory blood tests and clinical symptoms, 12 patients were proved to suffer from thyrotoxicosis [7–9, 11, 13, 15, 16, 18, 21, 30, 31, 33], and hyperthyroidism was seen in 13 cases. Most aspergillus thyroiditis cases showed signs in diagnostic imaging, but sonography and computed tomography presented nonspecific changes of thyroid gland. Histological tests (15 cases) and fine needle aspiration (15 cases) led to frequent diagnoses, but screening for aspergillus by detection of galactomannan was helpful in only 5 reports [17, 19, 21, 26, 29] (Table 1).

### Treatment and survival

Almost all of the patients received glucocorticoid and/or immunosuppressive agents, but only 10 patients [7, 11, 13, 14, 17, 18, 20, 24, 31, 33] suffered from neutropenia at the moment of aspergillosis diagnosis. Furthermore, only 2 of the neutropenia patients survived [17, 18]. Aspergillus thyroiditis appeared to be a high mortality disease, even under treatment by both antifungal drugs and surgery. Altogether, 10 patients survived [8, 9, 15, 17–19, 23, 25, 27, 29] and most of them were reported in the last couple of decades except one who survived in 1972 [9]. Six of the 10 patients had been treated with voriconazole with or without caspofungin or amphotericin B [8, 15, 17, 19, 27, 29], while only 2 of the survivors were successfully cured by amphotericin B monotherapy [9, 18]. It was remarkable that a total thyroidectomy without antifungal agents could also lead to survival in primary aspergillus thyroiditis excluding dissemination [25].

Probably many cases of genital involved disseminated aspergillosis were not reported, since it was a subset of cutaneous aspergillus infection. There were 7 reports of cutaneous and/or subcutaneous aspergillosis in the genital area identified in the literature [34–40]. Different from expectations, not all of the patients complained of skin lesions as the first clinical presentation. A variety of other symptoms were also revealed such as fever, perineum irritation, and difficulty in urination, defecation and sexual activity. Debridement was necessary and effective for most of the patients, while systemic antifungal administration was the cornerstone of successful treatment.

## Discussion and conclusion

Aspergillus species are ubiquitous and can be found in soil, dust, vegetation and decaying plant material [41]. It is the second most common cause of opportunistic fungal infection in humans after *Candida albicans*. It causes a severe infection in immunocompromised patients resulting in high mortality. Difficulty and delay in diagnosis and treatment often contributes to fatal outcomes. Commonly, it presents as a pulmonary infection, which invades the lung parenchyma and vasculature and later spreads to other organs. It has angioinvasive properties, which enables the fungus to disseminate via hematogenous spread.

The remarkable resistance to infection of the thyroid gland is due to its high iodine content, hydrogen peroxide production, abundant lymphatic and vascular supply, and its encapsulated location [42]. Postmortem studies have shown that thyroid aspergillus involvement constitutes 12% of extra pulmonary disease [43].

Whether aspergillus thyroiditis is an infrequent disease in populations remains to be established. In our case, the patient suffered from suppurative lesions due to aspergillus fumigatus both in the thyroid gland and subcutaneous tissue in the genital area. Although fungemia was never documented, the patient most likely had disseminated aspergillosis resulting from hematogenous spread. Pulmonary involvement had a strong possibility in this case considering of the radiological manifestations, despite the lack of a positive result from respiratory specimens. It is also possible that our patient contracted the infection from colonization of adjacent tissue, such as airway, related sinuses, and skin in the genital area.

The majority of aspergillus thyroiditis cases were asymptomatic and diagnosis was primarily classified at postmortem. In fact, local signs and symptoms of fungal thyroiditis are indistinguishable from symptoms of other infectious thyroiditis. Clinical diagnosis of aspergillus thyroiditis during life continues to be a major challenge. Fine needle aspiration cytology and culture have been the most frequent and successful diagnostic tests for detecting aspergillus thyroiditis ante mortem. It also plays an important role in the diagnosis of aspergillosis involving skin and soft tissue, which could difficult to distinguish by the naked eye.

Early diagnosis of aspergillosis and the establishment of aggressive therapy before more widespread dissemination of the infection likely contributed to the successful treatment in this patient. A satisfactory outcome of invasive aspergillosis is predicated on the return of normal bone marrow function and the prompt starting of appropriate antifungal agent therapy systemically as soon as the diagnosis is established. Treatment should include the judicious use of surgical intervention as clinical circumstance may indicate. As far as our patient was concerned, drainage was effective and remained an integral component of therapy for resolution of the aspergillus abscess.

Phagocytes, particularly neutrophils, play a critical role in the host’s defense against aspergillus. Studies have demonstrated that the incidence of invasive aspergillosis is directly related to the duration of neutropenia [44]. For disseminated disease, a reduction of immunosuppression can also help [5].

Amphotericin B was the mainstay of treatment for aspergillus infections even up to the 1990s, although nephrotoxicity limited its use and its efficacy was poor, especially in disseminated disease [45, 46]. Advancements in antifungal therapy have led to increased survival in patients with aspergillus infections. Voriconazole has better responses and improved survival compared to amphotericin B in invasive aspergillosis monotherapy [47], while caspofungin has been widely used as an effective ‘rescue’ therapy [48]. Combination medical therapy is a subject of great interest. The synergistic effect of voriconazole and caspofungin against aspergillus is supposed to involve the simultaneous inhibition of cell membrane and cell wall biosynthesis. This suggests that a combination of voriconazole and caspofungin might reduce mortality in critically ill patients.

Aspergillus thyroiditis typically begins with a brief hyperthyroidism phase due to the release of thyroid hormone as a result of follicular cell damage. Then transient euthyroidism ensues, usually followed by hypothyroidism that ultimately recovers to euthyroidism. Therefore, the thyroid function test can show variability [5]. Thyroid hormone levels ranged from those characteristic of hyperthyroidism to those typical of hypothyroidism. The management of thyroid hormone dysregulation is less often reported in the literature than the antifungal profile. The majority of our reviewed cases did not report the use of oral thyroid medications, and 2 cases reported that thyroid function could be normalized within 2 weeks following the initiation of antifungal therapy [9, 19]. This finding suggests that symptomatic treatment is sufficient for most patients due to the lack of thyroid-related symptomatology, even with laboratory evidence of thyroid dysfunction.

Invasive aspergillosis is a relatively frequent fungal infection occurred in immunocompromised patients, but aspergillus thyroiditis has been rarely reported. Aspergillosis involving the thyroid gland produces a high mortality rate > 60%, despite updated reports of patients treated with a novel azole, voriconazole, and an echinocandin, caspofungin. Early diagnosis of aspergillosis is a key to successful treatment. The clinicians must maintain a high level of diagnostic suspicion among those high-risk patients who present with fever and findings localized to the thyroid region or skin as well as soft tissue. Careful and thorough examinations are probably more important in those patients rather than subjective complaints, since a number of patient conditions are asymptomatic in the initial stage of invasive aspergillosis.

## References

[1] Barchiesi F, Mazzocato S, Mazzanti S, Gesuita R, Skrami E, Fiorentini A, Singh N (2015). Invasive aspergillosis in liver transplant recipients: epidemiology, clinical characteristics, treatment, and outcomes in 116 cases. Liver Transpl.

[2] Denning DW (1996). Therapeutic outcome in invasive aspergillosis. Clin Infect Dis.

[3] Vogeser M, Haas A, Ruckdeschel G, von Scheidt W (1998). Steroid-induced invasive aspergillosis with thyroid gland abscess and positive blood cultures. Eur J Clin Microbiol Infect Dis.

[4] Bartlett JG (2000). Aspergillosis update. Medicine.

[5] Nguyen J, Manera R, Minutti C (2012). Aspergillus thyroiditis: a review of the literature to highlight clinical challenges. Eur J Clin Microbiol Infect Dis.

[6] Gowing NF, Hamlin IM (1960). Tissue reactions to aspergillus in cases of Hodgkin's disease and leukaemia. J Clin Pathol.

[7] Badawy SM, Becktell KD, Muller WJ, Schneiderman J (2015). Aspergillus thyroiditis: first antemortem case diagnosed by fine-needle aspiration culture in a pediatric stem cell transplant patient. Transpl Infect Dis.

[8] Chung S, Lee JH, Lim Y, Yang HK, Chang YS (2010). Isolated aspergillus thyroiditis in an immunocompromised patient. NDT plus.

[9] Halazun JF, Anast CS, Lukens JN (1972). Thyrotoxicosis associated with aspergillus thyroiditis in chronic granulomatous disease. J Pediatr.

[10] Keane WM, Potsic WP, Perloff LJ, Barker CF, Grossman RA (1978). Aspergillus thyroiditis. Otolaryngology.

[11] Kishi Y, Negishi M, Kami M, Hamaki T, Miyakoshi S, Ueyama J, Morinaga S, Mutou Y (2002). Fatal airway obstruction caused by invasive aspergillosis of the thyroid gland. Leuk Lymphoma.

[12] Lisbona R, Lacourciere Y, Rosenthall L (1973). Aspergillomatous abscesses of the brain and thyroid. J Nucl Med.

[13] Marui S, de Lima Pereira AC, de Araujo Maia RM, Borba EF (2014). Suppurative thyroiditis due to aspergillosis: a case report. J Med Case Rep.

[14] Santiago M, Martinez JH, Palermo C, Figueroa C, Torres O, Trinidad R, Gonzalez E, Miranda Mde L, Garcia M, Villamarzo G (2013). Rapidly growing thyroid mass in an immunocompromised young male adult. Case Rep Endocrinol.

[15] Solak Y, Atalay H, Nar A, Ozbek O, Turkmen K, Erekul S, Turk S (2011). Aspergillus thyroiditis in a renal transplant recipient mimicking subacute thyroiditis. Transpl Infect Dis.

[16] Torres AM, Agrawal S, Peters S, Khurana K, Feiglin D, Schroeder E, Izquierdo R (1999). Invasive aspergillosis diagnosed by fine-needle aspiration of the thyroid gland. Thyroid.

[17] Zwitserloot AM, Warris A, van't Hek LG, van Die LE, Verweij PE, Mavinkurve-Groothuis AM (2008). Disseminated aspergillosis in an adolescent with acute lymphoblastic leukemia. Pediatr Blood Cancer.

[18] Kim SH, Kim JY, Park WC, Kim MK, Kim TJ (2016). Sequential sonographic features of primary invasive aspergillosis involving only the thyroid gland: a case report and literature review. Iran J Radiol.

[19] Ataca P, Atilla E, Saracoglu P, Yilmaz G, Civriz Bozdag S, Toprak SK, Yuksel MK, Ceyhan K, Topcuoglu P (2015). Aspergillus thyroiditis after allogeneic hematopoietic stem cell transplantation. Case Rep Hematol.

[20] Cornet M, Ugo V, Lefort E, Molina T, James JM, Vekhoff A, Audouin J, Marie JP, Bouvet A (2001). A case of disseminated aspergillosis with thyroid involvement. Eur J Clin Microbiol Infect Dis.

[21] Hornef MW, Schopohl J, Zietz C, Hallfeldt KK, Roggenkamp A, Gartner R, Heesemann J (2000). Thyrotoxicosis induced by thyroid involvement of disseminated aspergillus fumigatus infection. J Clin Microbiol.

[22] Matsui Y, Sugawara Y, Tsukada K, Kishi Y, Shibahara J, Makuuchi M (2006). Aspergillus thyroiditis in a living donor liver transplant recipient. J Infect.

[23] Thada ND, Prasad SC, Alva B, Pokharel M, Prasad KC (2013). A rare case of suppurative aspergillosis of the thyroid. Case Rep Otolaryngol.

[24] Winzelberg GG, Gore J, Yu D, Vagenakis AG, Braverman LE (1979). Aspergillus flavus as a cause of thyroiditis in an immunosuppressed host. Johns Hopkins Med J.

[25] Erdem H, Uzunlar AK, Yildirim U, Yildirim M, Geyik MF (2011). Diffuse infiltration of aspergillus hyphae in the thyroid gland with multinodular goiter. Indian J Pathol Microbiol.

[26] Elzi L, Laifer G, Bremerich J, Vosbeck J, Mayr M (2005). Invasive apergillosis with myocardial involvement after kidney transplantation. Nephrol Dial Transplant.

[27] Guetgemann A, Brandenburg VM, Ketteler M, Riehl J, Floege J (2006). Unclear fever 7 weeks after renal transplantation in a 56-year-old patient. Nephrol Dial Transplant.

[28] Solary E, Rifle G, Chalopin JM, Rifle-Mediavilla C, Rebibou JM, Camerlynck P, Justrabo E, Cuisenier B, Caillot D, Mousson C (1987). Disseminated aspergillosis revealed by thyroiditis in a renal allograft recipient. Transplantation.

[29] Cicora F, Mos F, Paz M, Roberti J (2013). Successful treatment of acute thyroiditis due to aspergillus spp. in the context of disseminated invasive aspergillosis in a kidney transplant patient. Nefrologia.

[30] Jang KS, Han HX, Oh YH, Paik SS (2004). Aspergillosis of the thyroid gland diagnosed by fine needle aspiration cytology. Acta Cytol.

[31] Alvi MM, Meyer DS, Hardin NJ, Dekay JG, Marney AM, Gilbert MP (2013). Aspergillus thyroiditis: a complication of respiratory tract infection in an immunocompromised patient. Case Rep Endocrinol.

[32] Sion ML, Armenaka MC, Georgiadis I, Paraskevopoulos G, Nikolaidis I (2004). Aspergillus fumigatus abscesses of the thyroid with obstruction of the esophagus. Thyroid.

[33] Ayala AR, Basaria S, Roberts KE, Cooper DS (2001). Aspergillus thyroiditis. Postgrad Med J.

[34] Arikan S, Uzun O, Cetinkaya Y, Kocagoz S, Akova M, Unal S (1998). Primary cutaneous aspergillosis in human immunodeficiency virus-infected patients: two cases and review. Clin Infect Dis.

[35] Chen Z, Li HM, Han W, Sang JH, Du J, Zhang WJ, Zhang JZ (2009). Genital cutaneous lesions in an allogeneic haematopoietic stem-cell transplant recipient with aspergillosis. Clin Exp Dermatol.

[36] Davido HT, Ryndin I, Kohler TS, Hadegard W, Monga M, Fung L (2007). Aspergillosis of the scrotum: non-surgical management. Int J Urol.

[37] Powell CR, Allshouse M, Bethel KJ, Mevorach RA (1998). Invasive aspergillosis of the scrotum. J Urol.

[38] Raszka WV, Shoupe BL, Edwards EG (1993). Isolated primary cutaneous aspergillosis of the labia. Med Pediatr Oncol.

[39] Li BK, Wang X, Ding Q (2009). A case report of severe aspergillus flavus penile infection. Asian J Androl.

[40] Tahir C, Garbati M, Nggada HA, Yawe EH, Abubakar AM (2011). Primary cutaneous aspergillosis in an immunocompetent patient. J Surg Tech Case Rep.

[41] Walsh TJ, Pizzo PA, Hoeprich PD, Jordan C, Ronald AR (1994). Aspergillosis. In: infectious diseases. 5th edn.

[42] Pearce EN, Farwell AP, Braverman LE (2003). Thyroiditis. N Engl J Med.

[43] Hori A, Kami M, Kishi Y, Machida U, Matsumura T, Kashima T (2002). Clinical significance of extra-pulmonary involvement of invasive aspergillosis: a retrospective autopsy-based study of 107 patients. J Hosp Infect.

[44] Saral R (1991). Candida and Aspergillus infections in immunocompromised patients: an overview. Rev Infect Dis.

[45] Denning DW, Stevens DA (1990). Antifungal and surgical treatment of invasive aspergillosis: review of 2,121 published cases. Rev Infect Dis.

[46] Ostrosky-Zeichner L, Marr KA, Rex JH, Cohen SH (2003). Amphotericin B: time for a new “gold standard”. Clin Infect Dis.

[47] Herbrecht R, Denning DW, Patterson TF, Bennett JE, Greene RE, Oestmann JW, Kern WV, Marr KA, Ribaud P, Lortholary O (2002). Voriconazole versus amphotericin B for primary therapy of invasive aspergillosis. N Engl J Med.

[48] Maertens J, Raad I, Petrikkos G, Boogaerts M, Selleslag D, Petersen FB, Sable CA, Kartsonis NA, Ngai A, Taylor A (2004). Efficacy and safety of caspofungin for treatment of invasive aspergillosis in patients refractory to or intolerant of conventional antifungal therapy. Clin Infect Dis.

